# Oral Microbiome in Oral Cancer Research from Sampling to Analysis: Strategies, Challenges, and Recommendations

**DOI:** 10.3390/cancers18010145

**Published:** 2025-12-31

**Authors:** Kelly Yi Ping Liu, Andrew Huang, Catherine Pepin, Ya Shen, Phoebe Tsang, Catherine F. Poh

**Affiliations:** 1 Department of Oral Biological and Medical Sciences, Faculty of Dentistry, University of British Columbia, 2199 Wesbrook Mall, Vancouver, BC V6T 1Z3, Canadaahhuang@student.ubc.ca (A.H.); yashen@dentistry.ubc.ca (Y.S.); phoebe.tsang.is@gmail.com (P.T.); 2 Department of Basic and Translational Research, BC Cancer Research Institute, 675 West 10th Avenue, Vancouver, BC V5Z 1L3, Canada; catherinepepin08@gmail.com; 3 School of Biomedical Engineering, University of British Columbia, 6088 University Boulevard, Vancouver, BC V6T 1Z3, Canada

**Keywords:** oral microbiome, oral cancer, dysbiosis, methodology, sampling, storage, sequencing, analysis pipeline, clinical applicability

## Abstract

The oral microbiome has become an emerging focus of oral cancer research, with growing evidence linking microbial communities to disease development, progression, and prognosis. However, there is limited consensus on optimal sampling strategies, storage methods, and analytical approaches. This narrative review critically evaluates current strategies for sampling, preservation, DNA extraction, sequencing, and data analysis in oral microbiome research related to oral cancer. We compared commonly used sampling methods, including saliva, oral rinse, swab, brush, and tissue biopsy, and reviewed preservation conditions, extraction kits, sequencing platforms, and analytical pipelines reported in recent oral microbiome studies. Sampling approaches affect microbial yield and site specificity. Saliva and oral rinse samples are convenient and noninvasive but may dilute lesion-specific microbial signals, whereas lesion-directed swabbing or brushing yields greater microbial biomass and biological relevance. Preservation media and storage temperature significantly influence microbial stability, and DNA extraction methods vary in their ability to remove host DNA. Although 16S rRNA gene sequencing remains the most common approach, shotgun metagenomics offers higher resolution and function insights but is still limited by clinical applicability. Differences in data pre- and post-processing models and normalization strategies further contribute to inconsistent microbial profiles. Given that oral mucosal sites differ markedly in structure and microenvironment, careful consideration is required to ensure that collected samples accurately represent the biological question being addressed. Methodological consistency across all workflow stages—from collection to analysis—is essential to generate reproducible, high-quality data and to enable reliable translation of oral microbiome research into clinical applications for cancer detection and risk assessment. Together, these insights provide a framework to guide future study design and support the development of clinically applicable microbiome-based biomarkers.

## Simple Summary

The human mouth contains many types of bacteria, the balance of which can shift, leading to “dysbiosis”, which may contribute to the development of oral cancer. Emerging studies are beginning to uncover the role of the oral microbiome and microbial changes in oral cancer risk and progression. However, current research methods vary widely, making it difficult to compare results across studies or identify reliable biomarkers for clinical applications. This review evaluates the different ways oral microbiome samples are obtained, including saliva, mouth rinse, swabs, and tissue, and how these choices affect findings. We highlight how DNA extraction and sequencing methods can introduce bias. We also emphasize the need for standardization and validation protocols, along with transparent reporting, to reduce methodological variability and enable reproducible, clinically interpretable oral microbiome data.

## 1. Introduction

The human oral cavity is home to one of the most diverse and dynamic microbial ecosystems in the body, consisting of bacteria, fungi, archaea, and protozoa. Collectively known as the oral microbiota, these communities play essential roles in maintaining oral and systemic health through metabolic, immunomodulatory, and protective functions [1,2]. Disruption of this ecosystem, known as dysbiosis, has been implicated not only in oral diseases such as dental caries, periodontal disease, and oral cancer [3,4], but also in a wide range of systemic conditions, including cardiovascular disease, diabetes, respiratory infections, rheumatoid arthritis, adverse pregnancy outcomes, and even neurodegenerative disorders [5,6,7,8,9].

Although many risk factors for oral cancer, such as tobacco and alcohol, are well established, a substantial proportion of cases arise in individuals without these exposures, suggesting additional etiologic factors. The oral microbiome has emerged as one such potential contributor, with studies linking specific microbial signatures to chronic inflammation, immune modulation, epithelial barrier disruption, and carcinogenic metabolites [10,11]. Shifts in microbial communities may precede or accompany malignant transformation, creating opportunities for early detection and risk stratification. Characterizing the oral microbiome in oral cancer could therefore provide new insights into disease mechanisms and support the development of microbiome-based diagnostic and prognostic tools.

Beyond taxonomic profiling, emerging multi-omic and functional approaches are increasingly used to interrogate microbial function and biological activity. These technologies enable researchers to examine not only which organisms are present, but also their metabolic pathways and interactions with epithelial and immune responses. Despite growing interest in the oral microbiome as a biomarker for oral cancer, there remains a limited consensus on standardized methodologies for sample collection, processing, and analysis. Such inconsistencies have hindered progress and validation of oral microbiome research.

The purpose of this narrative review is to provide a comprehensive methodological evaluation that can guide the design of future oral microbiome research in premalignant oral diseases and oral cancer. We examine how variations in biosample types, collection methods, preservation conditions, DNA extraction procedures, sequencing strategies, and data processing workflows influence microbial profiles. Our aim is to identify the major sources of technical variability that may limit reproducibility across studies and hinder the discovery of robust clinically applicable biomarkers.

### Review Design/Literature Search Strategy

This narrative review gathers recent evidence on sampling, preservation, DNA extraction, sequencing strategies, and data analysis in oral microbiome research related to oral premalignant lesions and oral cancer. We conducted targeted literature searches in PubMed and Web of Science for English-language articles published up to 30 August 2025 using combination of the following keywords: “oral microbiome”, “oral cancer”, “oral squamous cell carcinoma”, “oral potentially malignant lesions”, “sampling”, “saliva”, “rinse”, “swab”, “brush”, “tissue”, “DNA extraction”, “16S rRNA”, “shotgun metagenomics”, “pipeline”, and “database”. Additional relevant studies were identified by screening reference lists of key articles and recent reviews. Studies were included if they were relevant to our review objectives, with a focus on the methodology. Only human studies were included. The titles and abstracts of all articles were reviewed, and those relevant to our goals were selected for a full-text review. After reviewing the full texts, articles that were relevant were included in various sections.

## 2. Sample Types and Collection Techniques

Differences in sample types and collection techniques can lead to variability in microbial biomass, taxonomic composition, and downstream analysis. Therefore, the type of samples should depend on the research goal. In hypothesis-driven studies, where the goal is to test specific microbial-associated diseases, the target community can often be reasonably anticipated, and sampling can be tailored accordingly. In contrast, explorative studies that aim to broadly characterize community composition should prioritize representativeness and reproducibility to minimize technical bias. In oral cancer research, most microbiome-oriented studies remain exploratory, seeking to uncover potential microbial biomarkers. Consequently, variability in sampling techniques has become a major methodological consideration. Understanding these differences is essential for interpreting existing findings and for guiding standardization efforts. Table 1 provides an overview of current studies investigating the oral microbiome in the context of oral malignant and premalignant oral lesions with different sampling methodologies [12,13].

### 2.1. Saliva

Saliva is the most commonly collected oral sample in microbiome studies due to its ease of collection, noninvasiveness, and relatively high DNA yield. Since oral surfaces are bathed in saliva, these samples capture microbes from mucosal surfaces as well as free-floating bacteria, providing a broad overview of the oral microbiota. Unstimulated saliva is collected naturally through drooling, spitting, or cotton absorption [12]. This approach avoids stimulation-induced changes but may yield low volumes in patients with hyposalivation, such as those undergoing or recovering from oral cancer treatments, which can potentially alter microbial composition and diversity. In contrast, stimulated saliva, obtained by chewing paraffin or gum, tasting sour substances, or even stimulating imagery [12], provides larger sample volumes in a shorter time. While several studies have reported comparable results between stimulated and unstimulated saliva [24,25], others have documented compositional differences [26].

Saliva collection requires minimal technical expertise; therefore, it is suitable for self-administration and large-scale population studies. Moreover, the salivary microbiome has been shown to be relatively stable over short periods—from hours to weeks—and even up to a year [27,28]. Intra-individual profiles of both diversity and community composition remain more similar within a person than between individuals, with inter-individual differences dominating the variance [28]. Short-term, high-frequency sampling has revealed eating-associated fluctuations in specific taxa [29]. In contrast, in hospitalized or medically ill patients exposed to intensive chemotherapy and prolonged antibiotic use, repeated saliva sampling shows pronounced temporal instability. In addition, greater intra-patient variability is associated with adverse outcomes [30]. Together, these data suggest that saliva is a suitable method for temporal microbiome profiling within individuals, provided that sampling is standardized with respect to time of day, diet, and oral hygiene, and should account for medical conditions. In oral cancer research, saliva has been used to detect general microbial shifts or inflammatory signatures, but may be less informative for detecting early, localized changes in the mucosal microbiome, which are more effectively captured through site-directed sampling [17,19,31].

### 2.2. Oral Rinse

Oral rinse, similar to saliva, is an easy and noninvasive approach for collecting a broad, non-site-specific overview of the oral microbiome. The technique typically involves participants rinsing and/or gargling a defined volume of sterile collection medium or mouthwash for a fixed duration before spitting into a sterile tube. The rinsing process mechanically dislodges microorganisms from mucosal surfaces. In most studies, participants are instructed to alternate between swishing and gargling in five-second intervals for a total of 30 s before spitting the sample into a sterile container [32,33]. An alternate protocol involves swishing without gargling, such as the one described by Yu et al. [34]. Swishing alone may provide a more targeted profile of the oral cavity rather than the oropharyngeal microbiome [35]. While the choice of collection medium may influence taxonomic composition, mouthwash-based media have been shown to yield reliable oral microbiome profiles [33,35].

### 2.3. Exfoliation by Swabbing, Brushing, and Scrapping

Swabbing, brushing, and scraping enable the collection of site-specific microbial communities by targeting microbes attached to specific epithelial surfaces rather than free-floating in saliva. Swabbing is minimally invasive and typically performed with cotton or nylon-flocked swabs. Brushing, which uses cytobrushes or sponge brushes, is a more aggressive technique that dislodges biofilms and exfoliates cells. While both methods are effective for collecting niche-representative samples, brushing is generally preferred for lesion sampling as it yields higher microbial biomass and can recover microorganisms embedded deeper within biofilms [19]. In a study investigating the progression of oral epithelial dysplasia, researchers used cytology brushes (Innovatek Inc., Delta, BC, Canada) to exfoliate cells directly from oral lesions and obtained informative microbiome results [20]. Comparable success with brushing techniques has been demonstrated in other mucosal sites, such as the cervix and lung, where they provide high-quality microbial DNA for sequencing [36,37,38,39]. However, brushing may introduce higher proportions of host epithelial cells and can cause discomfort in sensitive tissues.

Scraping with a metal spatula is a more invasive method but provides enough material for microscopic analysis of epithelial-associated microorganisms, especially when samples are spread directly onto glass slides. This method enables fluorescent in situ hybridization (FISH) with DNA or RNA probes that target specific 16S rRNA sequences, allowing direct detection of microorganisms adhering to the epithelial cells [40,41]. When combined with sequencing data, this method can reveal the presence, relative abundance, and proximity of specific taxa to host cells, providing complementary insights into host–microbe interactions at the mucosal surface [40].

### 2.4. Biopsy Tissue Samples

Tissue samples are particularly valuable in oral cancer research as they capture microorganisms in direct association with precancerous or cancerous epithelium, potentially revealing site- or depth-specific microbial patterns not detectable with other sampling methods. Beyond enabling spatial localization of microbes within the tissue architecture, biopsy-based microbiome studies have identified unique bacteria associated with chronic inflammation and different clinical stages of cancer [15,17]. Similar to scraping samples, biopsies can be analyzed using FISH with probes specific to microbial antigens [42,43], allowing the visualization of microorganisms within the tissue microenvironment. This approach facilitates investigation of host–microbe interactions, localization of specific taxa, and validation of sequencing results. However, the use of biopsy samples in microbiome research is limited by clinical availability, ethical considerations, and sample size limitations.

In summary, the choice of sampling method influences the biological signal captured because different oral niches harbour distinct microbial communities. Saliva and oral rinses primarily reflect the pooled, planktonic microbiome, representing bacteria shed from multiple mucosal and dental surfaces. These non-site-specific samples are well-suited for population-level screening, epidemiologic studies, and assessing global microbial shifts, but they may dilute or mask localized dysbiosis associated with early lesions. In contrast, swabs and brushing samples adhere to biofilm directly, providing a more localized view of the microbial community. Lesion-directed brushing or swabbing enhances the detection of biomarkers associated with dysplasia, inflammation, or tumour-associated microbiota, making them more appropriate for risk stratification, early detection, and tumour microenvironment studies. Tissue biopsies offer the most spatially resolved information, enabling analysis of bacteria that physically associate with epithelial layers, invasion, or co-localize with inflammatory infiltrates. Thus, tissue samples are most suitable for studying host–microbe interactions, depth-specific ecology, and tumour-associated communities.

## 3. Sample Preservation and Storage Condition

Collected samples are usually stabilized using preservation media to prevent bacterial outgrowth, DNA degradation, and loss of microbial integrity. Effective preservation should minimize changes in microbial composition between collection and downstream processing. A variety of preservation media have been used for oral microbiome samples (Table 2). However, relatively few studies have directly compared their effects on microbial stability and sequencing outcomes. Recognizing and controlling for these influences is essential for producing reliable and comparable data [44,45].

### 3.1. Commercial Stabilization Kits

Commercial kits, such as OMNIgene ORAL (DNA Genotek Inc., Ottawa, ON, Canada) and DNA/RNA Shield (Zymo Research, Tustin, CA, USA), contain proprietary buffers designed to lyse host cells and preserve microbial DNA at room temperature. These kits are available in multiple formats, including saliva and site-specific sampling devices, and are widely used in oral microbiome research. While no universal “gold standard” exists for oral sample preservation, OMNIgene saliva kits are frequently used as a benchmark in comparative studies. According to the manufacturer’s data, DNA stability can be maintained up to 21 days, whereas swab-based kits can stabilize samples up to 30 days at room temperature [49]. The long stability period, combined with ease of use, makes commercial kits attractive for large cohort studies, multi-site collaboration, or remote and self-collection scenarios. However, comparative data between commercial kits remain limited.

### 3.2. Fixative-Type Preservation Media

Fixatives such as Saccomano’s fixative are designed to preserve cellular morphology but may alter microbial composition. The fixative, containing 50% ethanol and 2% Carbowax, was originally developed for sputum cytology and later adapted for microbiome studies [50]. While effective at maintaining cellular morphology, comparative studies show that samples preserved in Saccomanno’s fixative produce microbial profiles that differ from those obtained with other preservation methods, exhibiting increased beta-divergence, reduced alpha diversity, and altered taxonomic composition compared to commercial kits or ethanol-based oral rinses [35]. These differences are likely due to the relatively low ethanol concentration and the absence of dedicated microbial stabilizers, which permit bacterial overgrowth during storage, compromising the accuracy of microbiome profiling [35].

Another transport medium, the liquid dental transfer medium (LDTM), contains buffered mineral salts, sodium thioglycolate, and cysteine to maintain microbial viability while minimizing overgrowth during transport [51]. While OMNIgene samples typically show higher within-sample diversity [32], LDTM has demonstrated stability across a range of storage temperatures, making it a practical option for studies involving shipping or delayed processing. However, comparative studies show that LDTM samples harbor significantly different community compositions compared with OMNIgene samples, highlighting concerns for buffer-specific bias.

### 3.3. Ethanol- and Non-Ethanol-Based Mouth Washes

Compared with commercial kits and fixatives, ethanol and non-ethanol mouthwashes offer convenient and low-cost alternatives for sample storage. However, their chemical properties can influence the stability and accuracy of downstream microbial profiles. While generally stable at room temperature, studies have reported that samples stored in ethanol-based mouthwash begin to exhibit shifts in microbial composition after extended storage. For example, one study found that after four days at room temperature, *Firmicutes* increased while *Bacteroidetes*, *Proteobacteria*, and *Fusobacteria* decreased, relative to the sample preserved in OMNIgene [33]. In another study, 95% ethanol has been shown to preserve community profiles at room temperature for up to 8 weeks [52]. Although higher ethanol concentrations may improve stability, these findings have not been systematically validated for oral microbiome samples and should therefore be interpreted with caution.

Ethanol- and non-ethanol-based mouthwashes differ in how they interact with microorganisms. Ethanol reduces microbial viability through protein denaturation and membrane permeabilization, but it does not kill or lyse most bacteria because their cell walls typically remain intact. Consequently, ethanol-tolerant taxa may survive and proliferate slowly during storage, introducing post-collection shifts in microbial composition.

In contrast, non-ethanol preservation buffers often contain surfactants and chemical stabilizers, such as SDS in OMNIgene, that rapidly disrupt cell membranes, inactivate microbial metabolic activity, and suppress post-collection overgrowth [35]. These formulations are therefore more effective at maintaining the true microbial profile and may provide more consistent results than ethanol-based solutions.

Regardless of the chosen medium, it is important that the type of transport or preservation solution be consistent across all samples to ensure that the observed microbial differences reflect disease states rather than technical artifacts.

### 3.4. Storage Conditions

Beyond the choice of collection buffer, sample storage conditions may be another factor influencing sample stability and the integrity of oral microbiome data. Short-term storage at room temperature or 4 °C is often unavoidable, but the degree of microbial change depends largely on the medium used. Current evidence suggests that with appropriate preservatives (e.g., OMNIgene, DNA/RNA Shield), oral samples can remain stable at ambient temperature for 1–2 weeks [53]. Nonetheless, best practice from broader microbiome research recommends rapid freezing at −20 °C, and ideally −80 °C, particularly for studies requiring high sensitivity or long-term storage. Repeated freeze–thaw cycles should be minimized, as they can degrade DNA and reduce recovery of low-abundance organisms [52,53,54,55].

## 4. DNA Extraction and Sequencing

### 4.1. Microbial DNA Extraction

The goal of microbial DNA extraction is to efficiently recover high-quality nucleic acids while minimizing contamination from host DNA. Oral samples contain a large proportion of human epithelial cells and saliva; therefore, extraction protocols must balance effective depletion of host DNA and effective microbial cell lysis. A wide range of extraction methods and commercial kits are available, each differing in how they disrupt the cells and isolate the DNA. For example, the QIAamp DNA Microbiome kit (Qiagen) combines mechanical disruption, such as bead-beating, with host DNA depletion and enrichment of bacterial DNA. In contrast, the PureLink Microbiome DNA purification kit (Thermo Fisher Scientific, Waltham, MA, USA) recovers both microbial and host DNA, though its performance and suitability for integrated host–microbiome analyses in oral samples have not yet been evaluated [55].

Comparative studies have shown that extraction protocols incorporating mechanical disruption, such as bead-beating or vortex-assisted lysis, yield higher DNA quantities and capture greater microbial diversity compared to enzymatic or chemical lysis alone, particularly bacteria with robust cell walls [56]. Therefore, careful consideration should also account for DNA extraction methods that may bias relative abundance estimates. New host-depletion techniques that aim to improve microbial biomass have emerged. For example, osmotic lysis combined with propidium monoazide treatment has been used to enrich microbial DNA in both fresh and frozen saliva [57,58,59].

### 4.2. Sequencing Strategies

#### 4.2.1. 16S rRNA Gene Sequencing Preparation

16S rRNA amplicon sequencing is one of the most common methods for characterizing the oral microbiome. This approach targets hypervariable regions (V1–V9) of the bacterial 16S rRNA gene via PCR amplification, with primer selection strongly influencing taxonomic resolution. For example, Zheng et al. found that the V1–V3 regions captured higher phylotype richness compared to the V3–V4 regions. On the other hand, Kool et al. found that V3–V4 and V4–V5 regions yielded more reproducible results across replicates, although their findings were based on a synthetic community [60,61]. While most oral cancer studies focus on V3–V4 or V4–V5 [15,62] regions rather than V1–V3 [20,63,64], there is no consensus on which hypervariable regions offer optimal coverage and resolution, as results also depend on the sequencing platform and downstream analysis (Table 3).

#### 4.2.2. Shotgun Metagenomic Sequencing Preparation

Shotgun metagenomic sequencing provides comprehensive profiling of the genetic repertoire of a sample, enabling both species- and strain-level identification and functional annotation. Ganly et al. used metagenomic shotgun sequencing on oral rinse samples from nonsmoking OSCC patients and demonstrated distinct taxonomic and metabolic signatures compared with nonsmoking controls, including enrichment of pathways involved in vitamin, heme, and carbohydrate metabolism [65]. Among OSCC patients, Liu et al. further characterized microbiome variation across different depths of invasion, revealing progressive shifts in bacterial composition and microbial functions associated with more invasive tumours. Unlike 16S rRNA amplicon sequencing, shotgun sequencing is not limited to primer selection bias and can simultaneously detect bacteria, fungi, archaea, and viruses. However, shotgun sequencing suffers from substantial host DNA contamination, adapter ligation bias during library preparation, uneven genome coverage, and taxonomic misclassification resulting from incomplete or inaccurate reference databases [59]. Strategies to reduce host contamination include selective lysis of host cells, enzymatic digestion of host DNA, or targeting highly methylated eukaryotic DNA to enrich the remaining microbial DNA [59,66].

Sequencing strategies determine both the taxonomic resolution and functional insights in oral microbiome research. 16S rRNA gene sequencing remains a widely used and cost-effective approach, requiring relatively less DNA (10–50 ng DNA per sample) and modest sequencing depth. It is supported by well-established bioinformatics pipelines with limitations on primer bias, reduced taxonomic resolution, and inability to directly infer microbial function [67]. In contrast, shotgun metagenomic sequencing requires more DNA materials (100–1000 ng DNA per sample) and deeper sequencing coverage but provides species-to-strain-level precision and enables direct functional annotation of genes and pathways [68]. Given that shotgun sequencing is considerably more expensive, many studies use 16S rRNA sequencing for the full cohort and perform shotgun sequencing only on a smaller subset of samples. This targeted approach allows researchers to validate the 16S-derived taxonomic patterns and obtain additional functional and strain-level insights that 16S cannot provide [69].

### 4.3. Sequencing Platform Selection

The choice of sequencing platform can also influence oral microbiome data, as technologies differ in read length, throughput, and error rate. Awareness of these differences is critical when comparing results across studies or integrating multiple datasets.

Currently, Illumina platforms remain the most widely used platform in oral microbiome research as they provide high throughput with low error rates at relatively low cost. The MiSeq system is commonly used for 16S rRNA amplicon sequencing, whereas the NovaSeq platform supports deeper shotgun metagenomic sequencing for large-scale studies [70,71]. Compared with older platforms, such as pyrosequencing, Illumina’s higher accuracy, greater throughput, and mature bioinformatics pipelines make it well-suited for taxonomic profiling and diversity studies [72].

Third-generation sequencing technologies, such as PacBio HiFi and Oxford Nanopore, generate long-read sequencing that enables full-length 16S rRNA gene sequencing and improved species- or strain-level resolution in human oral microbiome samples [73]. PacBio HiFi, in particular, has improved species-level assignment compared to short-read sequencing [73]. However, PacBio systems generally offer lower throughput and higher per-sample costs, limiting their feasibility for large cohorts. Studies have reported mixed results regarding diversity estimation, with some studies showing no significant differences compared with Illumina data, while others report altered alpha diversity and relative abundance profiles [74]. While Oxford Nanopore sequencing provides real-time, portable sequencing capabilities, the higher error rates can reduce taxonomic accuracy unless robust error-correction algorithms are applied.

Comparative studies suggest that while estimates of alpha diversity and taxonomic abundance can vary among platforms, overall biological conclusions are consistent across methods. Studies must therefore balance cost, accuracy, read length, and data compatibility when selecting a sequencing platform [75].

## 5. Data Processing and Analysis

High-throughput sequencing produces large and complex datasets that require careful processing to ensure that downstream analyses accurately reflect the true microbial community. Key steps typically include quality filtering, chimera removal, denoising or clustering of sequence variants, and taxonomic classification using curated reference databases. Differences in how these steps are implemented can lead to substantial variability in reported microbial diversity and composition. As summarized in Table 3, studies vary widely in their sequence processing and taxonomic assignment methods, underscoring the importance of harmonized data-processing pipelines to minimize analytical bias.

### 5.1. Quality Control, Denoising, and Clustering

Processing raw sequencing data into biologically interpretable features involves a series of structured computational steps. These steps determine how microbial abundance is represented and are critical for ensuring that downstream analyses accurately reflect the underlying community. Processing typically begins with quality control that removes technical artifacts and low-quality reads. This includes adapter and primer trimming, filtering sequences by quality scores or expected error thresholds, removing sequences of inappropriate length, and identifying and removing chimeric reads [76,77]. Tools such as QIIME2, Mothur, USEARCH, FASTQC, Trimmomatic, and Cutadapt are commonly used for these steps. For shotgun metagenomics, tools such as FastQC [78] are commonly used to generate visual summaries of read quality, while Trimmomatic and Cutadapt are used for adapter trimming and quality filtering based on Phred scores [77,79].

After quality filtering, sequences are processed into either amplicon sequence variants (ASVs) or operational taxonomic units (OTUs), which are the core units to represent microbial features in downstream analysis. ASV-based approaches, such as DADA2 and Deblur, use statistical error models to correct sequencing errors and infer exact biological sequences. These methods detect single-nucleotide differences and provide higher resolution and taxonomic precision. In contrast, OTU-based approaches, such as CD-HIT, USEARCH/VSEARCH, or UNOISE3, cluster sequences into groups based on a predefined similarity threshold (typically ≥ 97–99.8%). While widely used in earlier studies and useful for integrating legacy datasets, OTU clustering can identify fine-grained ecological differences between closely related taxa [80,81]. Following clustering, sequences are collapsed into a feature table that summarizes the abundance of each ASV and OTU across samples.

For shotgun metagenomic sequencing, an additional preprocessing step is required that removes human reads. Oral samples often contain a large proportion of host DNA, which can obscure microbial signals. Host depletion is performed by aligning reads against the human reference genome and retaining only unmapped reads. Tools such as KneadData, BWA [82], or newer host-contamination removal frameworks such as HoCoRT are widely used for this purpose [83,84].

### 5.2. Reference Database for Taxonomic Assignment

Taxonomic assignment is typically performed using reference databases. For 16S rRNA gene sequencing, curated databases align reads to known taxa. The Human Oral Microbiome Database (HOMD) [85] is frequently used in oral microbiome studies because it provides oral-specific taxonomy and curated reference sequences from cultured and uncultured species. Broader databases such as SILVA [81], RDP [80], or Greengenes2 [86] offer wider phylogenetic coverage across diverse environments but may lack depth or accurate annotation for some oral-specific taxa. Some studies leverage multiple databases for validation or to achieve better genus- or species-level resolution. Reporting the version of the reference database and assignment parameters is therefore crucial for reproducibility and cross-study comparability.

For shotgun metagenomics sequencing, taxonomic and functional annotation require a more complex workflow because of the greater volume and diversity of data. After initial quality control, reads are often assembled into contiguous sequences (contigs) using assemblers, such as MEGAHIT [87] or metaSPAdes [88], that are optimized for large metagenomic datasets. Benchmarking studies have shown that no single assembler is universally optimal. Performance varies depending on factors such as sequencing depth, sample complexity, and computational resources [89,90]. Modular frameworks such as MetAMOS [91] provide flexibility to evaluate multiple assembly and annotation pipelines, optimizing performance for a given dataset [92]. In some cases, analysis of unassembled reads is preferred, particularly for highly diverse or low-coverage samples where assembly quality may be limited. For the taxonomic classification of such reads, k-mer-based algorithms such as Kraken and its derivatives [93,94,95] have demonstrated high accuracy and computational efficiency.

### 5.3. Normalization and Data Transformation

Interpretation of sequencing data requires careful normalization to account for variation in sequencing depth and library size across samples. No single method is universally optimal, and each introduces unique assumptions and biases. Recent benchmarking studies suggest that using a consensus-based approach by applying multiple methods and focusing on reproducible signals strengthens the robustness of microbial profiles [96]. The simplest normalization converts raw counts into relative abundances, expressing each taxon as a percentage of total reads per sample. Although this approach remains widely used, it is limited by the compositional nature of microbiome data, where all taxa within a sample must sum to 100% [97]. Consequently, an apparent increase in the relative abundance of one taxa reflect a decrease in another, even if its absolute abundance has not changed. Such interdependence between taxa can create spurious correlations and distort downstream analyses [98]. To address this, compositional-aware methods are increasingly recommended because they account for these dependences and provide more reliable inferences [96].

### 5.4. Sampling Depth and Filtering

Sequencing depth varies considerably between samples and platforms, making normalization and filtering essential to ensure fair comparisons. Rarefaction is a common approach for standardizing sequencing depth across samples, where samples are subsampled to the lowest acceptable read depth within a study. This procedure reduces biases introduced by uneven sequencing, as samples with more reads capture more rare taxa [99]. Rarefaction curves can guide this process, with plateaus indicating that sequencing depth is sufficient to capture most microbial diversity. However, rarefaction also discards valid reads, reduces statistical power, and may increase false positives or negatives. Alternatively, model-based normalization methods, such as the negative binomial models in DESeq2, have been developed [100] for sparse microbial count data using the median-of-ratios method. More recent compositional-aware tools, including ALDEx2, ANCOM-BC, corncob, and songbird, further account for zero-inflation and compositional constraints, offering improved control of false discoveries in microbiome datasets [101,102,103]. These approaches adjust for both library size and biological variability without discarding data, thereby improving reproducibility and sensitivity, particularly useful when sample sizes are limited or when subtle microbial shifts are being investigated.

Filtering low-abundance or low-relevance features is another critical-step to reduce noise [104]. Such thresholds are often determined empirically, but a prevalence threshold of 10% (i.e., retaining taxa detected in at least 10% of samples) is commonly applied to ensure more robust and reproducible results [96]. Relative abundance thresholds, typically between 0.01% and 0.1% per sample, help to remove extremely rare taxa that could distort diversity or differential abundance analyses [104]. However, overly stringent thresholds risk discarding rare but biologically meaningful taxa.

## 6. Discussion

The oral microbiome is a dynamic and diverse ecosystem influenced by host factors, environmental exposures, and disease processes. Understanding and standardizing sampling strategies is critical for the growing number of studies investigating oral premalignant and OSCC. Saliva and oral rinses offer convenient, noninvasive options suitable for population-level screenings and for investigating systemic associations. However, these pooled samples may dilute site-specific microbial signals, limiting their ability to detect localized dysbiosis associated with early lesion development. In contrast, site-directed sampling methods such as exfoliation and biopsies are the most appropriate when the research question targets lesion-associated microbial shifts, though they are invasive and require greater technical expertise. Collectively, there is no single sampling approach that is universally superior; rather, the optimal method must align with the biological question in study design.

In addition, maintaining consistency in preservation media, DNA extraction methods, and sequencing workflows is essential to minimize technical biases. Standardized protocols and transparent reporting will be critical to improving reproducibility, enabling cross-study comparison, and translating oral microbiome profiles into clinically meaningful applications. Recent reviews have further emphasized how methodological choices can shape biological interpretation. For example, Zhi et al. used shotgun metagenomics on tissue-based samples and identified *Streptococcus*, rather than *Fusobacterium*, as a dominant genus associated with oral cancer, contrasting with findings from other saliva-based 16S rRNA studies [105]. Such discrepancies highlight how differences in sampling sites, sequencing platforms, and analytical pipelines can bias which taxa emerge as the significant biomarkers for OSCC, emphasizing the need for methodological standardization under the same research goals.

Another important aspect is that the oral cavity comprises multiple anatomically and functionally distinct niches [106]. Each site, influenced by factors such as oxygen availability, moisture, nutrient gradients, pH, and host immune interactions, supports a unique microbial community. Understanding the clinical relevance of these niches is essential in oral cancer research, where lesion-associated dysbiosis is often site-specific. For example, the rough, papillary surface of the tongue harbors a community enriched in anaerobes, whereas the gingival crevice, affected frequently by periodontal disease, supports a markedly different microbial profile [107]. Paired sampling of a lesion and its contralateral normal mucosa provides a powerful within-subject design that controls for inter-individual variability and helps to highlight microbial shifts characterized in the abnormal lesion site.

The oral cavity is also easily influenced by short-term behavioral and environmental factors; pre-collection protocols are therefore essential for minimizing variability [29,108,109]. Standard recommendations include avoiding eating, drinking, smoking, chewing gum, and performing oral hygiene for at least 60 min prior to sampling. Time of day should also be standardized when possible, as circadian and eating-associated microbial fluctuation can affect community composition. Clear instructions and consistent training of participants and clinicians help to reduce pre-analytical variability. Recommendations for contamination control include the use of sterile collection instruments, gloves, and single-use consumables. Consistent adherence to pre-collection and contamination control procedures helps ensure that observed microbial differences reflect true biological variation rather than extraneous technical artifacts. In addition, it is important to recognize that sampling approaches, particularly invasive biopsy, may influence a patient’s willingness to participate, thereby potentially limiting recruitment and reducing cohort size. Even though longitudinal and repeated sampling can capture microbial dynamics associated with disease progression, accounting for temporal variation remains challenging.

Quality control is fundamental for reliable oral microbiome research. Positive controls, such as synthetic microbial communities, environmental samples from participants, or in vitro microbial models, can verify the integrity of extraction and sequencing workflows [110]. Negative controls, such as reagent blanks or extraction controls, are equally important, especially in low-biomass oral samples [110,111]. Given that sequencing is highly sensitive and can amplify even small amounts of contaminant DNA, negative controls are critical for detecting and addressing contamination. Studies should therefore transparently report their DNA extraction methods and contamination control measures to ensure reproducibility and comparability across studies [110]. Host DNA contamination, particularly in oral lesion or brush samples, remains a significant challenge [112]. In addition to the current methods involving mechanical disruption and selective lysis of mammalian cells, emerging approaches aim to further increase microbial signal through microbial enrichment and hybridization kits to selectively bind and remove host DNA before library construction. However, no host-depletion strategy is entirely unbiased. Overly aggressive depletion can inadvertently remove microbial taxa with fragile cell walls or low biomass.

While most oral microbiome studies rely on DNA-based sequencing to infer community composition and potential function, emerging RNA-based and other functional omics approaches are beginning to reveal active microbial pathways and host–microbe interactions. These methods, which capture transcriptionally active or metabolically expressed signals, can provide more dynamic insights into the biological role of the microbiome. However, they remain technically challenging. The review by Hu et al. highlights the promise of advanced assay technologies (e.g., mass spectrometry, multiplex platforms, nanobiosensors) and AI-driven analytics for saliva-based biomarker detection, as well as the persistent challenges of standardization and biological variability [113]. Incorporating multi-omic approaches, such as integrating metagenomic, transcriptomic, metabolomic, and host genomic data, can deepen biological interpretation and help identify mechanistic links between microbial pathways and host responses.

Clinical translation requires a robust framework to connect microbial alterations, reliable metrics to identify reliable biomarkers, and interventional studies to test whether modifying the microbiome can alter outcomes. Thus, a rigorous study design is essential to move from observational associations to clinical applications.

## 7. Conclusions

The oral microbiome represents a promising frontier in understanding the pathogenesis and progression of oral premalignant lesions and OSCC. Emerging evidence suggests that microbial signatures may serve as biomarkers for early detection, risk stratification, and monitoring treatment response. However, the translational potential of these findings remains constrained. Our review highlights key areas of inconsistency across studies, ranging from sampling and preservation methods to DNA extraction, sequencing strategies, and bioinformatics processing, that contribute to divergent microbial profiles and hinder reproducibility. Addressing these challenges through standardized and transparent methodologies will be critical to generate reliable and comparable data. Ultimately, oral microbiome profiling holds the potential to deliver noninvasive, clinically actionable biomarkers for early cancer detection and surveillance, as well as to identify therapeutic targets for microbiome modulation aimed at improving patient outcomes.

## Figures and Tables

**Table 1:** Sample types and sampling techniques in studies on oral precancers and cancer.

Study (Year)	Sampling Method(s)	Sample Size	Study Goal	Key Findings
Sasaki et al., 2005 [14]	Tissue & dental plaque	46	Compare plaque and tissue in OSCC patients vs. controls	Tissue and plaque in OSCC show distinct microbial community shifts
Pushalkar et al., 2012 [15]	Tissue	20	Profile tumour-associated microbiome in OSCC	OSCC tissues enriched in anaerobes like *Fusobacterium nucleatum*
Yang et al., 2018 [16]	Oral rinse	248	Analyze oral rinse differences between OSCC and controls	Significant changes in community composition in OSCC group
Yang et al., 2021 [17]	Saliva (unstimulated) & tissue	42	Characterize OSCC-associated changes in saliva and tissue samples	Tumour tissues enriched with anaerobes including *Fusobacterium nucleatum*
Gopinath et al., 2021 [18]	Saliva, swabbing, & biopsy tissue	94	Compare microbiomes across niches in oral cancer patients	Surface vs. deep tissue niche differences, and saliva-tissue comparisons in terms of composition and metabolic pathways
Shitozawa et al., 2022 [19]	Rinse & swabbing	10	Compare intra-individual differences of microbiota between lesion and normal and swabbing, vs. oral rinse	Lesion-specific dysbiosis observed, highlighting importance of site-specific sampling
Wright et al., 2023 [20]	Brushing & swabbing	90	Compare lesional vs. normal site microbiome in OSCC cases	Lesional sites showed enrichment of *Fusobacterium* and reduced diversity vs. contralateral controls
Mäkinen et al., 2023 [21]	Saliva (stimulated)	236	Survey salivary microbiome in OSCC vs. controls	Salivary profiles showed distinct community shifts in OSCC, with increased pathogenic taxa
Deo et al., 2025 [22]	Saliva	50	Compare normal, OPMD, OSCC microbiomes	Gram-negative and periodontal taxa increased in OSCC; unique taxa found in OSCC group
Intini et al., 2025 [23]	Saliva	66	Differentiate OL, PVL, OSCC microbiomes	OL shows higher richness, OSCC lowest, beta diversity distinguishes OL from PVL/OSCC

Abbreviation(s): OSCC, oral squamous cell carcinoma; OPMDs, oral potentially malignant disorders; OL, oral leukoplakia; PVL, proliferative verrucous leukoplakia.

**Table 2:** Oral microbiome studies comparing preservation buffer among different sampling techniques.

Study (Year)	Collection Method	Preservation Buffer/Kit	Sample Size	Key Findings
Luo et al., (2016) [32]	Plaque, saliva, swabbing	OMNIgene ORAL, Liquid Dental Transfer Medium (LDTM)	10	OMNIgene yielded higher diversity than LDTM; OMNIgene stable up to 1 week at room temperature, LDTM stable across multiple temperatures but with different composition
Vogtmann et al., (2019) [33]	Rinse & saliva (Scope^®^ mouthwash)	Scope^®^ mouthwash, OMNIgene ORAL	100	Mouthwash samples remained stable at room temperature for 4 days. Microbial community profiles differed between methods.
Zhou et al., (2019) [46]	Dental plaque	75% ethanol vs. bead solution	20	Storage medium influenced microbial recovery more than extraction method; ethanol and bead solutions maintained community structure well
Yano et al., (2020) [35]	Rinse (saline)	OMNIgene ORAL, Scope^®^ mouthwash, non-ethanol rinse, Saccomano’s fixative	40	OMNIgene yielded distinct, stable profiles while Saccomano’s samples showed the greatest divergence in diversity and taxonomic composition
Bang et al., (2023) [47]	Saliva, rinse (saline)	DNA/RNA Shield, OMNIgene ORAL saliva kit	3	Oral rinses showed higher diversity; OMNIgene, DNA/RNA Shield and simple collections produced similar profiles; taxonomic differences were not significant
Yano et al., (2023) [48]	Oral rinse	Ethanol-free, ethanol-based mouthwash	40	Both stable up to 10 days; minor taxa shifts by formulation; similar composition overall

**Table 3:** Sequencing strategies in oral microbiome studies of precancer and cancer.

Study (Year)	Sequencing Platform; 16S rRNA Region	General Processing Pipeline (Clustering)	Taxonomic Assignment	Depth (Reads)	Rarefied	Key Findings
Pushalkar et al., 2012 [15]	Sanger sequencing; V4–V5	Greengenes NAST (OUT)	HOMD	~95	No (rarefaction only)	Tumour tissues are enriched with anaerobes including *Fusobacterium nucleatum* and *Prevotella* species
Yang et al., 2021 [17]	Illumina MiSeq; V3–V4	DADA2 (ASV)	RDP, HOMD	~23,645	Not reported	Tumour and saliva showed distinct shifts. *Fusobacterium* is enriched in tumour tissue
Gopinath et al., 2021 [18]	Illumina MiSeq; V3–V5	DADA2 (ASV)	HOMD	Not reported	Not reported	Tumour surface enriched in Prophyromonas, *Fusobacteria*, and *Neisseria*. Deeper tissue enriched in *Prevotella* and *Treponema*. Saliva reflected tissue composition but had higher metabolic pathway diversity
Shitozawa et al., 2022 [19]	Sanger sequencing; V3–V5	CD-HIT-EST (OTU)	RDP	~192	No (rarefaction only)	Lesion microbiota is less diverse and distinct from contralateral sites. Oral rinses captured broader but less site-specific diversity
Mäkinen et al., 2023 [21]	Illumina MiSeq; V4	UNOISE3 (ASV)	HOMD	Not reported	Not reported	Post-treatment saliva showed reduced diversity but persistence of pathogenic taxa associated with OSCC.
Wright et al., 2023 [20]	Illumina MiSeq; V1–V3	Cutadapt, UNOISE3 (ASV)	HOMD	~7907	Yes	OEDs showed distinct microbial profiles with reduced diversity and enrichment of pro-inflammatory taxa

Abbreviation(s): ASV, amplicon sequence variant; OTUs, operational taxonomic units; HOMD, Human Oral Microbiome Database; RDP, the Ribosomal Database Project; OED, oral epithelial dysplasia; OSCC, oral squamous cell carcinoma.

## Data Availability

This review did not generate any new data.

## Abbreviations

The following abbreviations are used in this manuscript:

OSCC	Oral squamous cell carcinoma
OPMD	Oral potentially malignant disorders
OL	Oral leukoplakia
PVL	Proliferative verrucous leukoplakia
FISH	Fluorescence in situ hybridization
LDTM	Liquid dental transfer medium
ASV	Amplicon sequence variant
OTU	Operational taxonomic units
HOMD	Human Oral Microbiome Database
RDP	Ribosomal Database Project
OED	Oral epithelial dysplasia
